# Perioperative Glycemic Changes Following Partial Pancreatectomy for a Pancreatic Islet Cell Neoplasm in a Dog: A Case Report

**DOI:** 10.3390/vetsci13101108

**Published:** 2026-10-17

**Authors:** Cheol-Han Choi, Jae-Woo Kim, Min-Seok Park, Jun-Sik Cho, Dong-Hwi You, Jae-Hwan Kim, Ki-Dong Eom, Hwi-Yool Kim, Jung-Moon Kim

**Affiliations:** 1 Department of Veterinary Surgery, College of Veterinary Medicine, Konkuk University, 120, Neungdong-ro, Gwangjin-gu, Seoul 05029, Republic of Korea; cmfkdl@konkuk.ac.kr (C.-H.C.); jwkjwk97@naver.com (J.-W.K.); kafa9913@gmail.com (M.-S.P.); junsik10@naver.com (J.-S.C.); dongwhi1016@naver.com (D.-H.Y.); hykim@konkuk.ac.kr (H.-Y.K.); 2 Department of Veterinary Medical Imaging, College of Veterinary Medicine, Konkuk University, 120 Neungdong-ro, Gwangjin-gu, Seoul 05030, Republic of Korea; jaehwan@konkuk.ac.kr (J.-H.K.); eomkd@konkuk.ac.kr (K.-D.E.)

**Keywords:** canine insulinoma, partial pancreatectomy, postoperative hyperglycemia, glycemic monitoring

## Abstract

Canine insulinomas are functional pancreatic neuroendocrine tumors that typically cause hypoglycemia, but blood glucose may fluctuate before surgery and increase after resection. This report describes the diagnostic evaluation, surgical treatment, and perioperative glycemic course of a 12-year-old female Miniature Schnauzer with a pancreatic islet cell neoplasm considered clinically consistent with insulinoma. Intermittent low or low-normal blood glucose concentrations ranging from 54 to 72 mg/dL were documented before surgery, and the blood glucose concentration on the day of surgery was 73 mg/dL. Contrast-enhanced computed tomography identified a mass in the left pancreatic limb, and partial pancreatectomy was subsequently performed. Blood glucose reached 416 mg/dL 2 h after pancreatic mass removal while surgery was ongoing, then initially declined without insulin administration. Recurrent postoperative hyperglycemia was treated with regular insulin on postoperative days (PODs) 2 and 3, whereas subsequent decreases in blood glucose were treated with dextrose supplementation. Blood glucose continued to fluctuate on POD 4; no additional insulin was administered after POD 3. Histopathologic examination identified a pancreatic islet cell neoplasm. Although a paired serum insulin concentration during documented hypoglycemia was not available and immunohistochemical staining for insulin or other neuroendocrine markers was not performed, the clinical, imaging, glycemic, and histopathological findings were considered consistent with a clinicopathologic diagnosis of insulinoma. Blood glucose was 150 mg/dL on POD 7; subsequent recorded concentrations through POD 150 ranged from 107 to 133 mg/dL. Serial glucose monitoring helped characterize the perioperative course and guide postoperative insulin and dextrose treatment.

## Simple Summary

Insulinoma is a tumor of the pancreas that produces excessive amounts of insulin and can cause low blood sugar in dogs. However, blood sugar levels may vary over time, which can make recognition of the disease more difficult. This report describes a 12-year-old Miniature Schnauzer with a pancreatic mass and intermittently low blood sugar levels. The mass was identified using computed tomography and surgically removed together with a small amount of surrounding pancreatic tissue. Blood sugar increased markedly after removal of the mass but initially decreased without insulin treatment. Recurrent high blood sugar during recovery was treated with short-term insulin, and subsequent decreases in blood sugar were treated with dextrose. Microscopic examination identified a pancreatic islet cell neoplasm. Although insulin production by the neoplastic cells was not directly confirmed, the overall findings were considered consistent with insulinoma. No additional insulin was administered through postoperative day 150. Blood sugar was measured repeatedly before, during, and after surgery to follow these changes.

## 1. Introduction

Insulinoma is a functional pancreatic neuroendocrine tumor arising from insulin-producing β-cells and is the most common endocrine pancreatic neoplasm in dogs [1,2,3,4]. Excessive insulin secretion results in hypoglycemia and may cause clinical signs including weakness, altered mentation, ataxia, tremors, collapse, and seizures [1,2,3,4,5]. Canine insulinomas are generally considered biologically malignant because of their high metastatic potential, with the liver and regional lymph nodes being common sites of metastasis [1,2,3,4].

The diagnosis of canine insulinoma is generally based on the presence of hypoglycemia with an inappropriately normal or increased circulating insulin concentration, together with compatible clinical findings [1,3,4]. However, blood glucose concentrations may fluctuate, and clinical signs can be intermittent [1,5]. Insulinoma has also been reported in a normoglycemic dog, indicating that the absence of hypoglycemia on a single measurement does not exclude the disease [6].

Preoperative localization of the pancreatic lesion is important for surgical planning. Abdominal ultrasonography and computed tomography (CT) have been used for the detection and localization of canine insulinomas [7,8]. Contrast-enhanced CT, including multiphasic protocols, can facilitate lesion localization [8,9,10]. Canine insulinomas commonly show arterial-phase enhancement, although their conspicuity varies among contrast phases [8,9,10].

Surgical treatment is generally preferred for dogs with resectable insulinoma and has been associated with longer survival than medical management alone [11,12,13]. Partial pancreatectomy is commonly performed for localized, surgically accessible pancreatic lesions [12,14]. Postoperative hyperglycemia may occur following surgical treatment and may require insulin therapy in some dogs [14,15,16].

This report describes the diagnostic evaluation, surgery, and perioperative glycemic course of a dog with a pancreatic islet cell neoplasm and findings consistent with insulinoma, emphasizing serial glucose monitoring after resection.

## 2. Case Presentation

**Figure 1:**
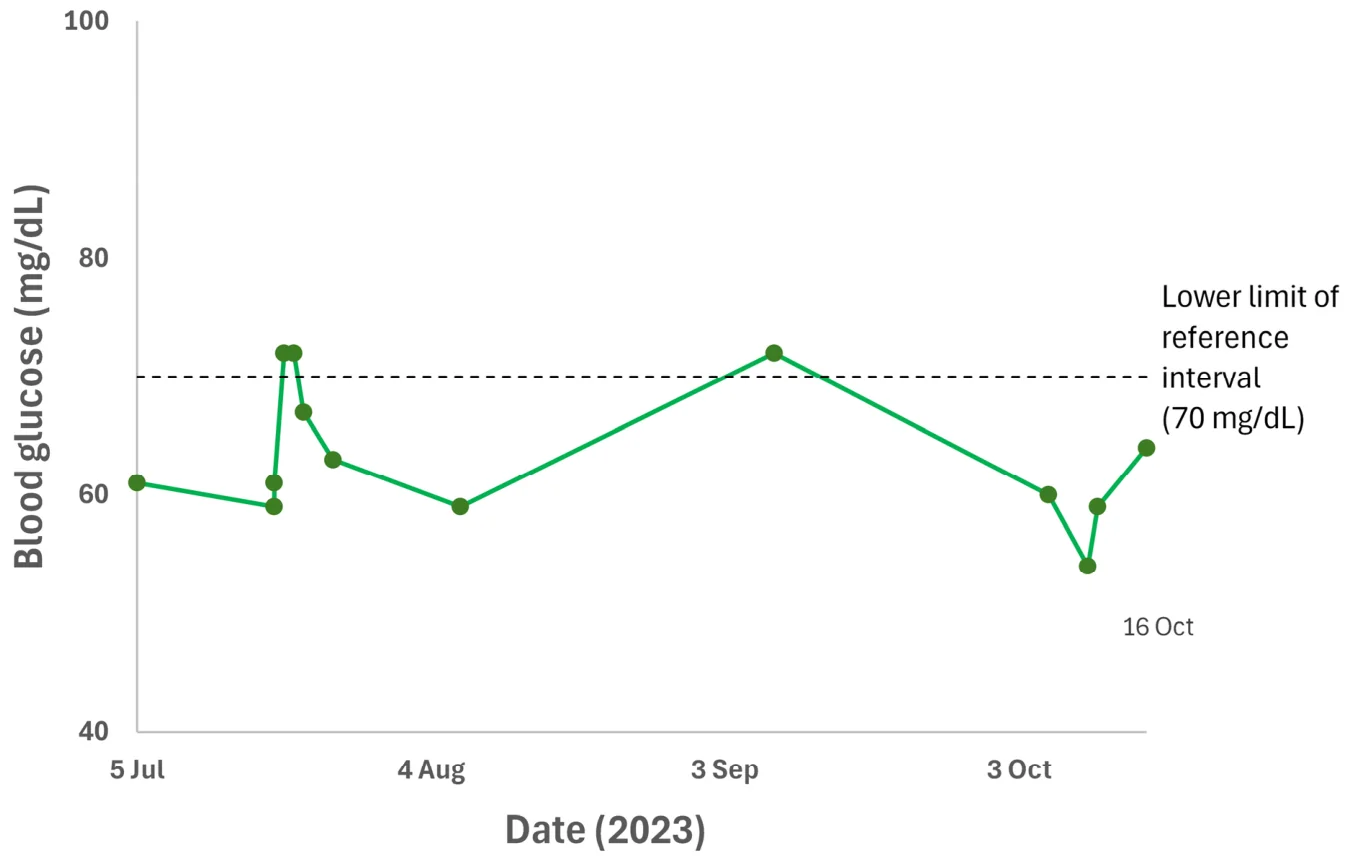
Preoperative blood glucose concentrations. Blood glucose concentrations measured from 5 July to 16 October 2023 are shown chronologically. All measurements were performed on centrifuged plasma using a Catalyst One or Catalyst Dx chemistry analyzer (IDEXX Laboratories, Inc., Westbrook, ME, USA). The horizontal dashed line marks the lower limit of the laboratory reference interval (70–143 mg/dL). Lines connecting data points aid visualization and do not represent continuous glucose monitoring.

A 12-year-old female Miniature Schnauzer weighing 7.4 kg was initially presented for evaluation of mammary gland masses. Physical examination identified eight mammary masses distributed across both mammary chains. Abdominal ultrasonography (V8; Samsung Medison, Seoul, Republic of Korea) performed as part of the initial evaluation identified two small hepatic nodules. Two weeks later, the dog underwent left unilateral mastectomy, ovariohysterectomy, cystotomy, removal of cutaneous masses, and inguinal hernia repair. During the approximately three-month period between the initial presentation and pancreatic surgery, intermittent low or low-normal blood glucose concentrations ranging from 54 to 72 mg/dL were documented (reference interval, 70–143 mg/dL; Figure 1). No overt clinical signs attributable to hypoglycemia were observed.

Approximately 3 weeks after the initial surgery, an adrenocorticotropic hormone (ACTH) stimulation test was performed as part of the evaluation for recurrent hypoglycemia. The baseline cortisol concentration was 4.4 μg/dL, and the post-ACTH cortisol concentration was 17 μg/dL (reference interval, 6–18 μg/dL). Routine complete blood counts and serum biochemical profiles were available during the preoperative evaluation. Mild elevations in alanine aminotransferase and alkaline phosphatase activities were recorded at the initial presentation, and transient leukocytosis and increased C-reactive protein concentrations were recorded after the earlier surgery. At the assessment with the lowest recorded preoperative blood glucose concentration of 54 mg/dL, hematocrit, leukocyte count, albumin, renal indices, sodium, potassium, alanine aminotransferase, and alkaline phosphatase were within their respective reference intervals.

**Figure 2:**
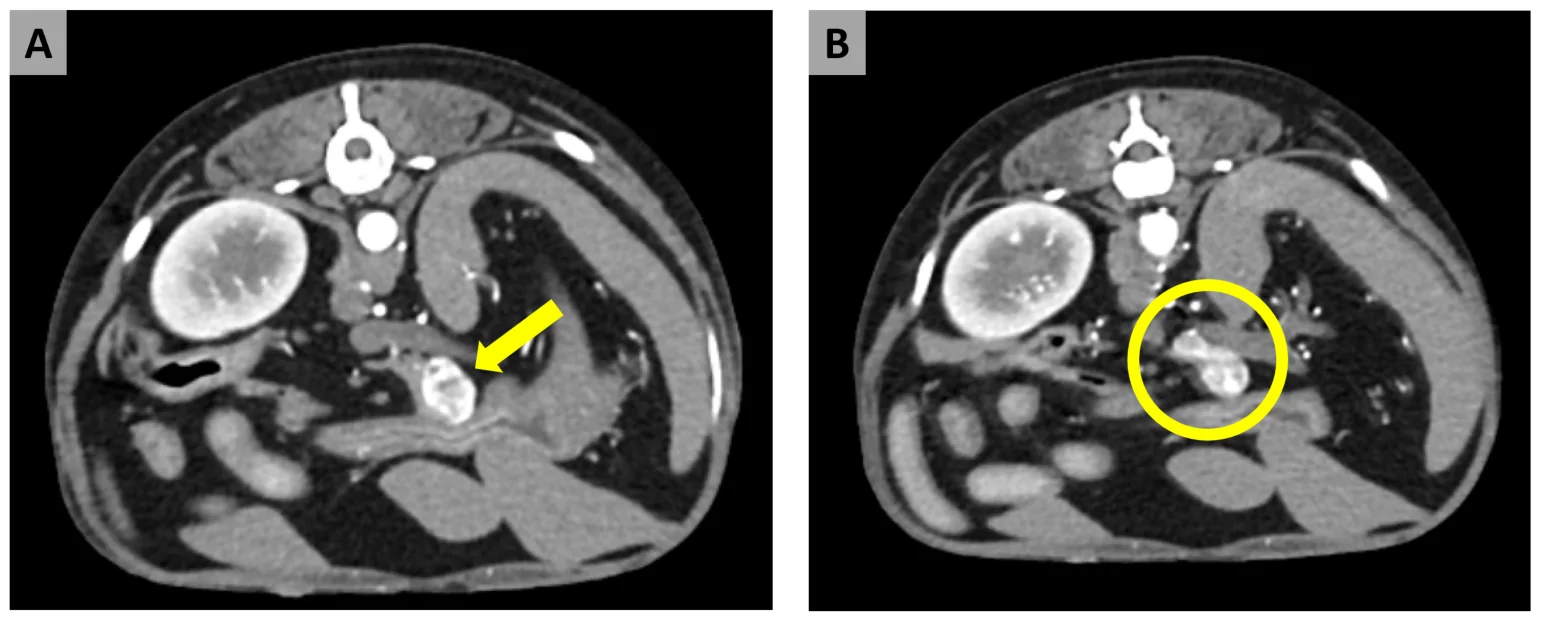
Arterial-phase axial contrast-enhanced computed tomography images of the pancreatic mass. (**A**) A mass in the left pancreatic limb showing marked contrast enhancement (yellow arrow). (**B**) The enlarged and contrast-enhancing left pancreatic limb containing the mass (yellow circle).

Six days before pancreatic surgery, contrast-enhanced CT performed during evaluation of the remaining mammary gland tumors identified a mass measuring 21.9 × 10.7 × 13.4 mm in the left pancreatic limb (Figure 2). Iohexol (Omnipaque 300; GE Healthcare, Chicago, IL, USA) was administered intravenously at 2 mL/kg at an injection rate of 2 mL/s. Images were acquired at 15 s (arterial phase), 30 s (pancreatic phase), and 90 s (equilibrium phase) after the start of contrast administration. The lesion was most conspicuous during the arterial phase. The mass showed strong heterogeneous contrast enhancement with marked capsular enhancement, and a feeding artery arising from the cranial mesenteric artery was identified.

No obvious intra-abdominal lymph node or parenchymal metastases were identified on CT. Considering the pancreatic mass together with the repeated low or low-normal blood glucose concentrations, insulinoma was suspected. A paired serum insulin concentration during documented hypoglycemia was not available.

Laboratory blood glucose concentrations were measured in centrifuged plasma using Catalyst One or Catalyst Dx chemistry analyzers (IDEXX Laboratories, Inc., Westbrook, ME, USA). Point-of-care concentrations were measured in paw-pad capillary whole blood using a FreeStyle Optium Neo handheld glucometer (Abbott Diabetes Care, Alameda, CA, USA). The preoperative glucose measurements and the 73 mg/dL measurement on postoperative day (POD) 0 used the laboratory plasma method; both laboratory and point-of-care methods were used during perioperative monitoring. The fasting status at the time of the preoperative blood glucose measurements was not consistently documented in the available medical records.

Preoperative blood glucose was measured intermittently, and perioperative measurement frequency varied according to the clinical course. During a closely monitored intraoperative period, glucose was measured at 5–18 min intervals. After each regular insulin administration, the first recheck was performed 15 min later, followed generally by measurements at approximately 30 min intervals during the initial glucose decline.

On the day of pancreatic surgery (POD 0), the dog consumed a small amount of low-fat canned food. A blood glucose concentration of 73 mg/dL was recorded before anesthesia. Later, before anesthetic induction, blood glucose was 47 mg/dL, and 35 mL of 10% dextrose prepared by diluting 50% Dextrose Injection (Dai Han Pharm. Co., Ltd., Seoul, Republic of Korea) was administered by slow intravenous injection over 5 min.

General anesthesia was induced with midazolam (0.2 mg/kg IV; Bukwang Midazolam Inj., Bukwang Pharm., Seoul, Republic of Korea) and propofol (4 mg/kg IV; Anepol Inj., Hana Pharm., Seoul, Republic of Korea) and maintained with isoflurane (Terrell Solution, Kyongbo Pharm., Asan, Republic of Korea). Plasma Solution A Inj. (HK inno.N, Cheongju, Republic of Korea) was administered as the baseline intravenous crystalloid fluid during anesthesia. Fentanyl (2 µg/kg IV per administration; Fentanyl Citrate Inj., Hana Pharm., Seoul, Republic of Korea) was given once before induction and once during surgery. Remifentanil (Remiva Inj., Hana Pharm., Seoul, Republic of Korea) was infused for intraoperative and early postoperative analgesia at 0.2 µg/kg/min, reduced to 0.1 µg/kg/min after surgery, and discontinued on POD 1. Cefazolin (22 mg/kg IV; Chongkundang Cefazolin Inj. 1 g, Chongkundang Pharm., Seoul, Republic of Korea) and famotidine (1 mg/kg IV; Gaster Injection, Dong-A ST Co., Ltd., Seoul, Republic of Korea) were also administered perioperatively.

**Figure 3:**
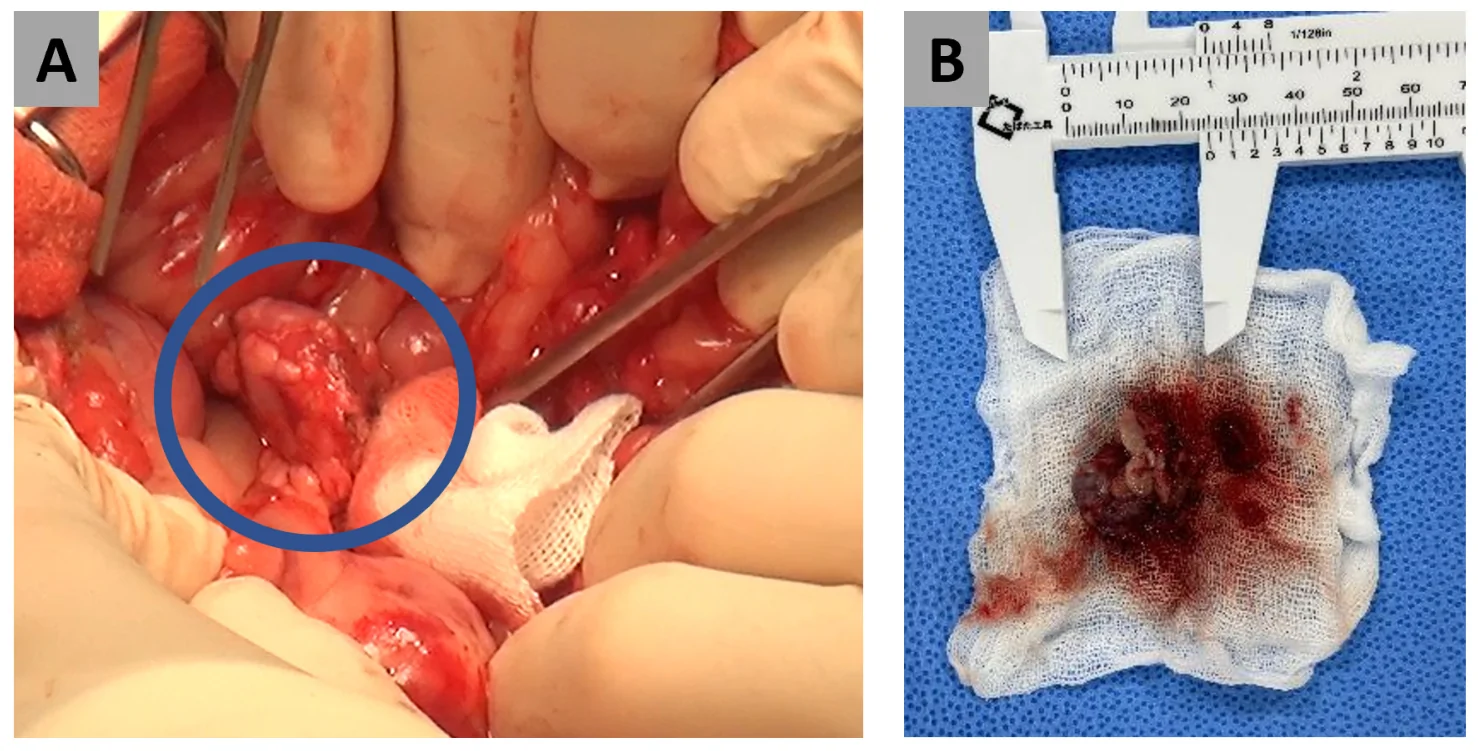
Intraoperative and gross appearance of the pancreatic mass. (**A**) The pancreatic mass identified in the left pancreatic limb during surgery (blue circle). (**B**) Gross appearance of the resected pancreatic mass with a ruler for scale.

At surgery, the mass was identified at the tip of the left pancreatic limb and appeared grossly distinct from the adjacent pancreatic tissue, without visible adhesions. Following blunt dissection of the surrounding tissue, partial pancreatectomy was performed using a vessel-sealing device (LigaSure Exact Dissector, LF2019; Medtronic, Minneapolis, MN, USA), with approximately 1 cm of grossly normal-appearing pancreatic tissue included in the resection (Figure 3). The liver and regional lymph nodes were inspected and palpated intraoperatively, and no gross abnormalities were identified. Neither hepatic tissue nor regional lymph nodes were sampled.

**Figure 4:**
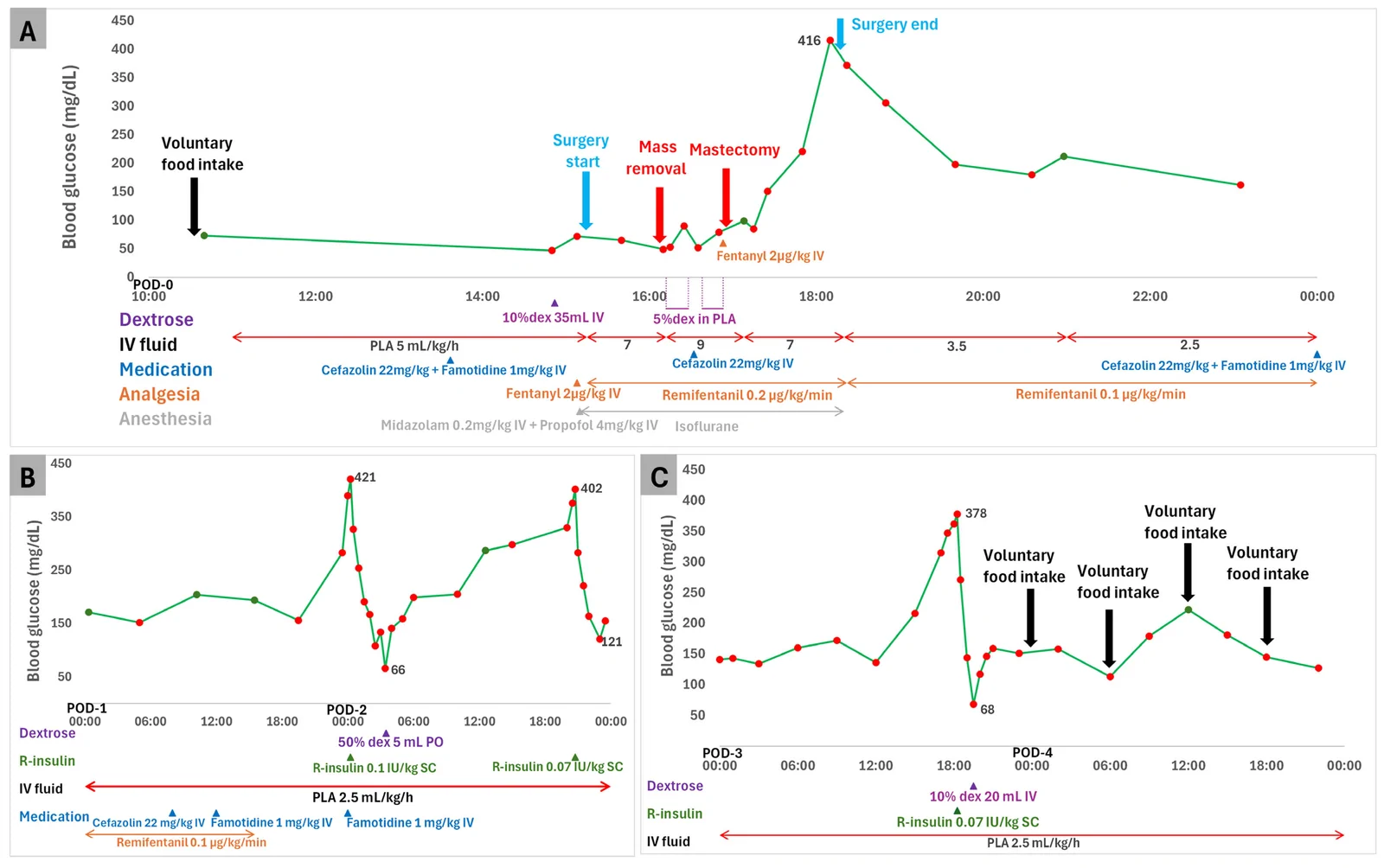
Perioperative blood glucose concentrations and recorded interventions. Green circles indicate measurements in centrifuged plasma using a Catalyst One or Catalyst Dx chemistry analyzer; red circles indicate point-of-care measurements in paw-pad capillary whole blood using a FreeStyle Optium Neo glucometer. (**A**) On the day of surgery (POD 0), the dog consumed a small amount of low-fat canned food at 10:30. Blood glucose was 47 mg/dL at 14:50, and 10% dextrose (35 mL) was administered by slow intravenous injection over 5 min at 14:52. Surgery began at 15:20, and the pancreatic mass was removed at 16:10, when blood glucose was 49 mg/dL. Plasma Solution A containing 5% dextrose was administered during two intervals, 16:12–16:28 and 16:38–16:53. Right unilateral mastectomy began at 16:55. Blood glucose reached 416 mg/dL at 18:10, before surgery ended at 18:20. Glucose initially declined without insulin administration. (**B**) PODs 1–2. Blood glucose rose overnight from 283 mg/dL at 23:30 on POD 1 to 390 mg/dL at 00:00 and 421 mg/dL at 00:15 on POD 2. Regular insulin (0.1 IU/kg SC) was administered at 00:15. Blood glucose decreased to 66 mg/dL, and 50% dextrose (5 mL) was administered orally at 03:30. Later on POD 2, glucose rose from 330 to 376 and then 402 mg/dL; a second dose of regular insulin (0.07 IU/kg SC) was administered at 20:45. The lowest recorded concentration after this dose was 121 mg/dL, and no dextrose was administered during that episode. (**C**) PODs 3–4. On POD 3, blood glucose rose on serial measurements from 315 to 347, 362, and 378 mg/dL. Regular insulin (0.07 IU/kg SC) was administered at 18:15. Blood glucose decreased to 68 mg/dL, and 10% dextrose (20 mL) was administered by slow intravenous injection over 5 min at 19:30. The first postoperative voluntary food intake was recorded at 00:00 on POD 4. The timelines beneath the glucose plots show the recorded fluid rates and interventions. Connecting lines aid visualization and do not represent continuous glucose monitoring. Horizontal arrows indicate periods of continuous administration, and triangles indicate individual administrations. In the figure labels, POD denotes postoperative day; PLA, Plasma Solution A; dex, dextrose; R-insulin, regular insulin; IV, intravenous; PO, per os; and SC, subcutaneous.

Blood glucose was 49 mg/dL at pancreatic mass removal. Following pancreatic mass removal, Plasma Solution A Inj. with 5% dextrose was administered intermittently, and right unilateral mastectomy began later during the same surgery. Blood glucose reached 416 mg/dL 2 h after mass removal, while surgery was ongoing. Glucose initially declined without insulin administration (Figure 4A).

Postoperatively, regular insulin was administered when blood glucose exceeded 360 mg/dL and continued to rise on serial measurements. Following regular insulin administration, blood glucose was monitored serially, and dextrose supplementation was given when blood glucose decreased below 70 mg/dL.

On POD 2, blood glucose rose from 390 to 421 mg/dL on serial measurements, with the 421 mg/dL concentration prompting the first dose of regular insulin (0.1 IU/kg SC; Humulin R Inj., Lilly Korea Ltd., Seoul, Republic of Korea)**,** after which glucose decreased to 66 mg/dL. Given this concentration and the overall post-insulin decline, 5 mL of a 50% dextrose solution formulated for injection was administered orally while the dog was alert and able to swallow (Figure 4B; Table 1). Later on POD 2, blood glucose rose from 330 to 376 and then 402 mg/dL on serial measurements, with the 402 mg/dL concentration prompting another dose of regular insulin. The dose was reduced to 0.07 IU/kg SC in view of the marked glucose decline after the initial dose. The lowest recorded glucose concentration during this treatment episode was 121 mg/dL, and no dextrose was administered (Figure 4B; Table 1).

On POD 3, blood glucose rose from 315 to 347, 362, and 378 mg/dL on serial measurements, with the 378 mg/dL concentration prompting another dose of regular insulin (0.07 IU/kg SC). Blood glucose subsequently decreased to 68 mg/dL, and 10% dextrose (20 mL IV) was administered by slow intravenous injection over 5 min (Figure 4C; Table 1). No additional insulin was administered after POD 3. Food was offered from POD 2, with the first voluntary intake recorded on POD 4 after the final insulin and dextrose administrations.

**Figure 5:**
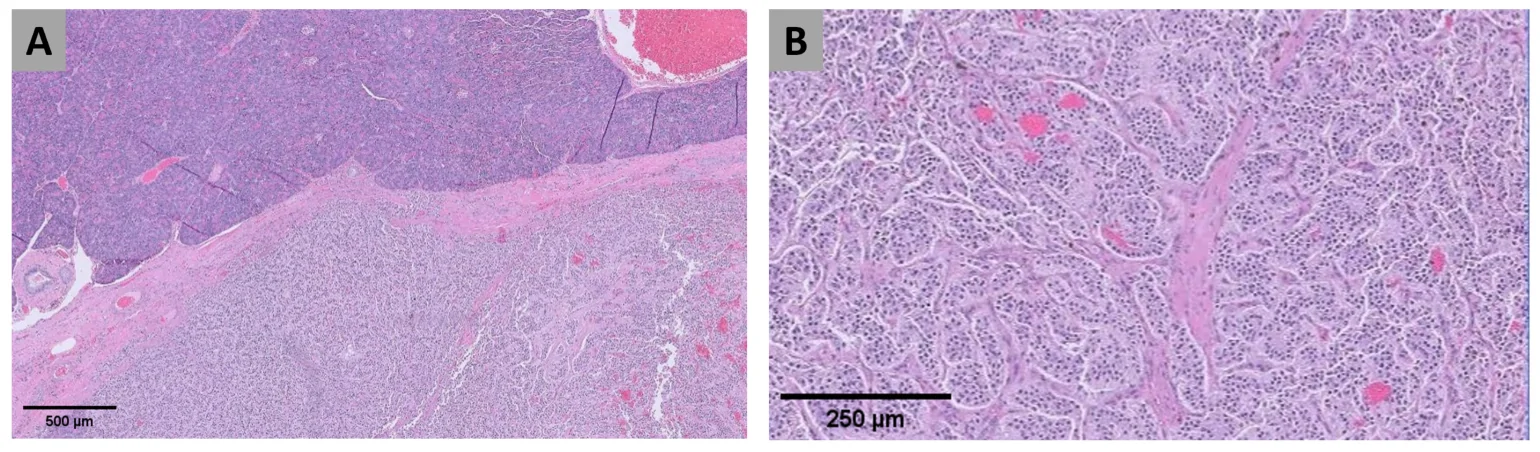
Histopathologic appearance of the pancreatic islet cell neoplasm. (**A**) Lower-power view showing the neoplasm and adjacent pancreatic tissue. (**B**) Higher-power view showing neoplastic cells arranged in lobules and packets separated by scant fibrovascular stroma. Hematoxylin and eosin staining. Scale bars = 500 μm (**A**) and 250 μm (**B**).

Histopathologic examination of the resected pancreatic lesion revealed a fairly well-demarcated proliferation of neoplastic neuroendocrine cells arranged in lobules and packets separated by scant fibrovascular stroma (Figure 5). The neoplastic cells were polygonal, with indistinct cell borders, eosinophilic wispy cytoplasm, round-to-ovoid finely stippled nuclei, variably prominent nucleoli, and moderate anisokaryosis. The mitotic count was 3 per 2.37 mm^2^. Vascular invasion was not identified, and the surgical margins were histologically clear but narrow, with the nearest peripheral margin measuring < 1 mm. Anisocytosis, necrosis, unequivocal invasion of surrounding tissues, and encapsulation were not specifically described in the original histopathology report. Therefore, the lesion was conservatively classified in this report as a pancreatic islet cell neoplasm.

Immunohistochemical staining for insulin or other neuroendocrine markers, including chromogranin A and synaptophysin, was not performed. Therefore, insulin expression by the neoplastic cells was not demonstrated. Together with the absence of a paired serum insulin concentration during documented hypoglycemia, these findings precluded unequivocal confirmation of a functional insulinoma. Nevertheless, the repeated low or low-normal blood glucose concentrations, pancreatic mass, perioperative glycemic course, and histopathologic findings were considered consistent with a clinicopathologic diagnosis of insulinoma.

On POD 7, blood glucose was 150 mg/dL, above the reference interval (70–143 mg/dL). Blood glucose was subsequently reassessed on POD 10, 13, 17, 23, 41, 48, 80, and 150; recorded concentrations ranged from 107 to 133 mg/dL without insulin administration. Follow-up abdominal ultrasonography performed approximately five months after surgery showed pancreatic thickness within the normal range, stable sublumbar lymph node size, and no significant interval change in the size or ultrasonographic appearance of either hepatic nodule.

## 3. Discussion

The present case was characterized by repeated low or low-normal blood glucose concentrations without overt clinical signs attributable to hypoglycemia. During the preoperative evaluation, recorded blood glucose concentrations ranged from 54 to 72 mg/dL, and the initial concentration on the day of pancreatic surgery was 73 mg/dL. These measurements included concentrations below or near the lower limit of the reference interval. This variability illustrates why an isolated glucose measurement may be insufficient when insulinoma is suspected [1,5,6]. In this dog, the pancreatic lesion was detected during evaluation for another disease, and the repeated low glucose concentrations prompted consideration of its possible functional significance. Because serum insulin was not measured during documented hypoglycemia, insulinoma could not be confirmed biochemically [1,3,4].

Other potential causes of the repeated low or low-normal blood glucose concentrations included clinically significant hepatic disease, sepsis, hypoadrenocorticism, severe systemic illness, and nonpancreatic neoplasia. Although mild elevations in hepatic enzyme activities and transient increases in leukocyte count and C-reactive protein were recorded earlier, routine laboratory findings at the assessment with the lowest preoperative glucose concentration of 54 mg/dL did not identify a clear alternative cause. The clinical significance of the two small hepatic nodules identified on initial ultrasonography remained undetermined. The adequate cortisol response to ACTH stimulation made hypoadrenocorticism unlikely. The mammary lesions were histopathologically consistent with benign mammary neoplasia. Nevertheless, the available evaluation could not definitively exclude other nonpancreatic neoplastic or systemic causes of hypoglycemia.

Contrast-enhanced CT localized the pancreatic lesion to the left pancreatic limb and showed strong heterogeneous enhancement with marked capsular enhancement. These findings were compatible with reported CT features of canine insulinoma [7,8,9,10]. No obvious intra-abdominal lymph node or parenchymal metastases were identified on CT, although microscopic metastatic disease could not be excluded.

Partial pancreatectomy was performed because the lesion was localized and considered surgically resectable. Surgical treatment is generally preferred for resectable canine insulinoma [1,11,12,13,14]. Because a standardized surgical margin for canine insulinoma has not been established, the gross resection distance described above should not be regarded as a recommended oncologic margin.

Although 10% dextrose had been administered before anesthetic induction, blood glucose was 49 mg/dL at pancreatic mass removal and reached 416 mg/dL 2 h later, while surgery was ongoing (Figure 4A). Intraoperative glucose increases have been reported in dogs undergoing surgery for insulinoma [16]. The timing was compatible with a reduction in insulin effect after pancreatic mass removal, but did not establish reduced insulin effect as the sole cause of the glucose increase. The preinduction 10% dextrose administration and subsequent intraoperative exposure to 5% dextrose-containing fluid may also have contributed to the magnitude of the increase. The concurrent right unilateral mastectomy added surgical stress that could have affected perioperative glycemia. The glycemic response cannot confirm complete removal of insulin-secreting tumor tissue or exclude residual or metastatic disease.

Hyperglycemia was treated with short-term regular insulin, whereas subsequent decreases in blood glucose were treated with dextrose supplementation. The use of short-term regular insulin in this case reflected an individualized clinical management decision and should not be interpreted as a recommendation for routine insulin treatment of postoperative hyperglycemia. No additional insulin was administered after POD 3, although glucose concentrations continued to fluctuate on POD 4. Follow-up without insulin treatment was compatible with a transient disturbance of glucose regulation; persistent postoperative diabetes mellitus was not documented. Postoperative hyperglycemia is recognized after surgical treatment of canine insulinoma [1,14,15,17]. Altered function of the remaining pancreatic β-cells following chronic hyperinsulinemia has been proposed as a contributor to postoperative changes in glucose regulation [1,14]. Pancreatic resection, anesthesia, and perioperative stress may also have affected glucose regulation in this dog.

Histopathologic examination identified a pancreatic islet cell neoplasm. A paired serum insulin concentration during documented hypoglycemia was not available, and immunohistochemical staining for insulin or other neuroendocrine markers was not performed. Therefore, insulin secretion during hypoglycemia and insulin expression by the neoplastic cells were not demonstrated, precluding unequivocal confirmation of a functional insulinoma. Although hormone expression and functional activity are not necessarily concordant in canine islet cell tumors [18], the clinical course supported insulinoma as the leading clinicopathologic diagnosis. Accordingly, this case is described as a pancreatic islet cell neoplasm with findings consistent with insulinoma rather than an unequivocally confirmed functional insulinoma.

No additional insulin was administered through POD 150. Because overt clinical signs attributable to hypoglycemia were not observed preoperatively, the absence of such signs during follow-up was of limited value for assessing recurrence. The available follow-up was insufficient to establish long-term oncologic disease control, as recurrence of hypoglycemia has been reported after surgical treatment of canine insulinoma [1,11,12,13].

The main limitations of this report were the absence of a paired serum insulin concentration during documented hypoglycemia, lack of insulin immunohistochemistry, potential confounding effects of preinduction and intraoperative dextrose administration and concurrent surgery on perioperative glucose concentrations, and the follow-up period through POD 150. Laboratory plasma and capillary whole-blood glucometer measurements are not necessarily directly interchangeable, particularly near treatment thresholds. These limitations reduce diagnostic certainty and restrict interpretation of the perioperative glycemic response and long-term oncologic outcome.

## 4. Conclusions

This case describes a pancreatic islet cell neoplasm with clinical, imaging, glycemic, and histopathological findings consistent with insulinoma. Glucose rose intraoperatively after pancreatic mass removal and fluctuated during early recovery; postoperative hyperglycemia was treated with short-term insulin therapy. Serial monitoring guided treatment, and interpretation of the glucose course should account for dextrose administration, insulin treatment, concurrent surgery, and perioperative stress.

## Figures and Tables

**Table 1:** Postoperative blood glucose concentrations and treatments associated with regular insulin administration.

Insulin Administration	Final Two Pre-Insulin BG Measurements (mg/dL)	Regular Insulin Dose and Route	Lowest Recorded Post-Insulin BG (mg/dL; Time After Insulin)	Dextrose Administration
POD 2, 00:15	00:00, 390 → 00:15, 421	0.1 IU/kg SC	66; 3 h 10 min	03:30; 50% dextrose, 5 mL PO
POD 2, 20:45	20:30, 376 → 20:45, 402	0.07 IU/kg SC	121; 2 h 15 min	None
POD 3, 18:15	18:00, 362 → 18:15, 378	0.07 IU/kg SC	68; 1 h 15 min	19:30; 10% dextrose, 20 mL IV over 5 min

Abbreviations: BG, blood glucose; POD, postoperative day; SC, subcutaneous; PO, per os; IV, intravenous.

## Data Availability

The original contributions presented in this study are included in the article. Further inquiries can be directed to the corresponding author.

## Abbreviations

The following abbreviations are used in this manuscript:

CT	Computed tomography
ACTH	Adrenocorticotropic hormone
POD	Postoperative day
IV	Intravenous
SC	Subcutaneous
PO	Per os
